# Effect of DHT-Induced Hyperandrogenism on the Pro-Inflammatory Cytokines in a Rat Model of Polycystic Ovary Morphology

**DOI:** 10.3390/medicina56030100

**Published:** 2020-02-27

**Authors:** Abhaya Krishnan, Sridhar Muthusami, Loganayaki Periyasamy, Jone A. Stanley, Vasudevan Gopalakrishnan, Ilangovan Ramachandran

**Affiliations:** 1 Department of Biochemistry, Karpagam Academy of Higher Education, Coimbatore 641 021, Tamil Nadu, India; abhayak.bio@gmail.com (A.K.); logabiochemistry@gmail.com (L.P.); 2 Department of Large Animal Clinical Sciences, Texas A&M University, College Station, TX 77843, USA; jstanley@cvm.tamu.edu; 3 Department of Endocrinology, Dr. ALM PG Institute of Basic Medical Sciences, University of Madras, Taramani Campus, Chennai 600 113, Tamil Nadu, India; gkibms@gmail.com (V.G.); ilangovan2000@yahoo.com (I.R.)

**Keywords:** DHT, PCOM, liver, ovary, bone, inflammatory cytokine

## Abstract

*Background and Objectives*: Polycystic ovary syndrome (PCOS) is one of the most prevalent disorders among women of reproductive age. It is considered as a pro-inflammatory state with chronic low-grade inflammation, one of the key factors contributing to the pathogenesis of this disorder. Polycystic ovary is a well-established criterion for PCOS. The present investigation aimed at finding the role of hyperandrogenism, the most important feature of PCOS, in the development of this inflammatory state. To address this problem, we adopted a model system that developed polycystic ovary morphology (PCOM), which could be most effectively used in order to study the role of non-aromatizable androgen in inflammation in PCOS. *Materials and Methods*: Six rats were used to induce PCOM in 21-days-old female Wistar albino rats by using a pre-determined release of dihydrotestosterone (DHT), a potent non-aromatizable androgen, achieved by implanting a DHT osmotic pump, which is designed to release a daily dose of 83 μg. *Results*: After 90 days, the rats displayed irregular estrous cycles and multiple ovarian cysts similar to human PCOS. Elevated serum inflammatory markers such as tumor necrosis factor-α (TNF-α) and interleukin-1β (IL-1β), and the presence of a necrotic lesion in the liver, osteoclast in the femur, multinucleated giant cells and lymphocytes in the ovary based on histopathological observation of DHT-treated rats clearly indicated the onset of inflammation in the hyperandrogenic state. Our results show no significant alterations in serum hormones such as luteinizing hormone (LH), follicle stimulating hormone (FSH), insulin, and cortisol between control and hyperandrogenised rats. DHT was significantly elevated as compared to control. mRNA studies showed an increased expression level of TNF-α and IL-1β, further, the mRNA expression of urocortin 1 (Ucn-1) was stupendously elevated in the liver of hyperandrogenised rats. *Conclusions:* Thus, results from this study provide: (1) a good PCOM model system in order to study the inflammatory changes in PCOS aspects, (2) alteration of inflammatory markers in PCOM rats that could be either due to its direct effect or by the regulation of various inflammatory genes and markers in the liver of hyperandrogenic state suggesting the regulatory role of DHT, and (3) alteration in stress-related protein in the liver of PCOM rats.

## 1. Introduction

Polycystic ovary syndrome (PCOS) is a complex disorder with endocrine and metabolic abnormalities seen in premenopausal women. A combination of ovarian dysfunction and excess androgenism is a common feature in PCOS [1]. Approximately 6–20% of women in reproductive age are predicted to suffer from PCOS [2,3]. PCOS women have multiple reproductive defects including hyperandrogenism, oligo-/an-ovulation, and multifollicular ovarian morphology [4]. The etiological factors for the onset of PCOS include epigenetic modifications, lifestyle, and environmental exposure to xenoestrogens/xenoandrogens. A previous review published from our laboratory revealed the link between various endocrine changes and its relevance to bone function in PCOS [5]. Polycystic ovary morphology (PCOM) is a well-established feature of PCOS-like phenotype, as PCOS is based on ovarian morphological changes associated with increased ovarian volume, ovarian area, hyperandrogenism and anovulation that collectively leading to reprogramming of hypothalamic-pituitary-ovarian axis [4,6].

Previous studies have documented that PCOS subjects are in a chronic pro-inflammatory state [7]. Serum and follicular fluid levels of interleukin-1β (IL-1β), interleukin-6 (IL-6) and tumor necrosis factor-α (TNF-α) are elevated in PCOS women [8,9]. The hypothalamus-pituitary-adrenal (HPA) axis, which plays a major role in adrenal steroidogenesis and metabolic factors including obesity, and insulin-related signals are found to be activated by inflammatory cytokines such as IL-1 [10,11]. The non-alcoholic fatty liver disease (NAFLD) is reported in PCOS subjects [12,13]. Hepatocytes are known to be the target of action of a variety of cytokines, and alteration in the activities is associated with various physiological and pathological states. Inflammation is also known to influence various aspects of liver development in addition to altering acute liver injury and fibrosis [14]. However, there is no evidence regarding the expression of cytokines, mainly IL-1β, IL-6 and TNF-α, in the liver of hyperandrogenised PCOM rat models.

A striking correlation exists between stress and progression of PCOS [15]. Urocortin (Ucn) belongs to the corticotrophin-releasing factor (CRF) family of peptides consisting of 41 amino acids [16], which are involved in many of the endocrine responses towards stress. Ucn-1 shares 45% sequence identity with rat/human CRF and Ucn-1 expression is mainly reported in the Edinger-Westphal nucleus (EWN) [16,17].

The present investigation is aimed at delineating the expression pattern of inflammatory cytokines TNF-α, IL-1β, and IL-6, as well as stress-related protein Ucn-1 in the liver of dihydrotestosterone (DHT)-treated PCOM rat models. To the best of our knowledge, the role of hyperandrogenism in the expression pattern of pro-inflammatory cytokines on the liver has not been reported. Collectively, these observations warranted an exploration of the regulatory role of hyperandrogenism in the expression pattern of inflammatory cytokines in the liver of female rats.

## 2. Materials and Methods

### 2.1. Preparation of DHT-Filled Osmotic Pumps

To stimulate hyperandrogenemia, the silastic tubes (I.D. 1.98 mm × O.D. 3.18 mm; Dow Corning, Midland, MI, USA) were used along with the implantable osmotic pump (Durect Corporation, Curpertino, CA, USA) filled with DHT as described earlier [18]. The osmotic pump releases the controlled, predetermined level of DHT (3.46 µg/h) based upon the osmotic pressure difference between the osmotic layer and the tissue environment.

### 2.2. Animal Surgery and DHT Osmotic Pump Implantation

The protocols employed in the present experiments were approved by duly constituted Institutional Animal Ethics Committee (IAEC), Karpagam Academy of Higher Education (AUP approval # KU/IAEC/Ph. D/155). Twelve female Wistar albino rats, 21 days old, were split into two groups (control and hyperandrogenised PCOM group), comprising 6 rats per each group. Control group rats were sham-operated and implanted with an empty osmotic pump filled with sterile vehicle. Hyperandrogenised PCOM group of rats were surgically implanted with a DHT-filled osmotic pump capable of releasing a total of 83 μg of DHT per day to stimulate the PCOM in rats that mimic the hyperandrogenic state in women with PCOS [19]. The DHT was administered to rats for a total of 90 days. The animals were monitored weekly to record the bodyweight. The estrous cycle phases of rats were monitored through the routine examination of vaginal smears for the presence of cornified cells. The animals were euthanized after 90 days of post-implantation and their ovaries, liver, femur, and blood were collected for further analyses.

### 2.3. Measurement of Serum Hormones

Blood samples were subjected to centrifugation at 3000 rpm for 15 min. The collected serum was stored at −80 °C for further analysis. Commercially available enzyme-linked immunosorbent assay (ELISA) kits were used (Cloud-Clone Corp) to determine the levels of serum follicle-stimulating hormone (FSH, Cat # CEA830Ra, assay sensitivity 2.47–200 ng/mL), luteinizing hormone (LH, Cat # CEA441Ra, assay sensitivity 98.77–8000 pg/mL), dihydrotestosterone (DHT, Cat # CEA443Ge, assay sensitivity 30.9–2500 pg/mL), cortisol (Cat # CEA462Ge, assay sensitivity 12.35–1000 ng/mL) and insulin (Cat # CEA448Ra, assay sensitivity 123.5–10,000 pg/mL) as per the manufacturer’s instruction. The intra-assay and inter-assay coefficient of variations were 10% and 12%, respectively.

### 2.4. Histopathology

#### 2.4.1. Decalcification of Femur

Femur bone was decalcified by removing mineral, other calcified tissue and preserving all the essential microscopic elements. Femurs were fixed in 4% paraformaldehyde with constant agitation at room temperature for 24 h and were rinsed in running tap water for 24 h. Then the femurs were incubated with decalcifying solution 10% EDTA (pH 7.4) for 21 days at 4 °C under continuous shaking. The solution was replaced every 24 h. It was then neutralized for 30 min using 0.1% aqueous ammonia solution. The samples were then kept at 4 °C under continuous shaking for complete decalcification [20].

#### 2.4.2. H&E Staining

H&E staining was performed for ovary, liver and decalcified femur following a routine procedure as described earlier [21]. After deparaffinization and rehydration, 5 μm sections were stained using hematoxylin solution for 5 min and by briefly placing them in 1% acid ethanol (1% HCl in 70% ethanol) and rinsing them with distilled water. This was followed by 3 min eosin staining and subjected to graded alcohol dehydration, and cleared using xylene. The processed slides were then analyzed with a light microscope and photographed.

### 2.5. Analysis of mRNA Expression (Semi-Quantitative PCR)

Total RNA was isolated from control and DHT-treated PCOM rat liver using the TRIzol reagent (monophasic solution of phenol and guanidinium isothiocyanate; Cat # 93289, Millipore Sigma, Burlington, MA, USA) at indicated time points. The purity and concentration of RNA were determined spectrophotometrically by measuring the absorbance at 260/280 nm; a purity of 1.8–2.0 proceeded for the PCR analysis. 100 ng of total RNA was mixed with the master mix containing RT-PCR buffer (10 μL), dNTP mix (2 μL), RT-PCR enzyme mix (2 μL), 3 μL of 10 μM (final concentration 0.6 μM) of sense and anti-sense primers (Table 1) and was reverse transcribed using one-step RT-PCR kit (Cat # 210210, Qiagen, Hilden, Germany) according to manufacturer’s instructions and further amplified by PCR. The cDNA was resolved in 2% agarose gel and visualized under UV light and documented using Chemidoc XRS+ imager (Bio-Rad, Hercules, CA, USA).

## 3. Statistical Analysis

The data were subjected to statistical analysis using one-way analysis of variance (ANOVA) and Duncan’s multiple range tests to assess the significance of individual variations between groups using the statistical analysis software SPSS 7.5 Students’ version (SPSS Inc., Chicago, IL, USA). The results were expressed as mean ± SD and *p* < 0.05 was considered statistically significant.

## 4. Results

### 4.1. Confirmation of PCOM

**Figure 1:**
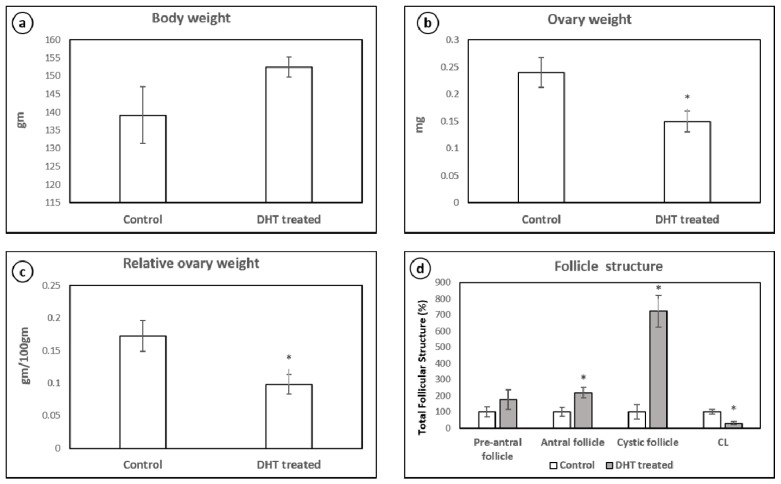
Body weight (**a**), total ovary weight (**b**), relative ovary weight (**c**) and follicle structure (**d**) in control and DHT-treated hyperandrogenised PCOM rat. Each bar represents mean ± SD (n = 6). ‘∗’ denotes statistical significance at *p* < 0.05 when compared with control.

**Figure 2:**
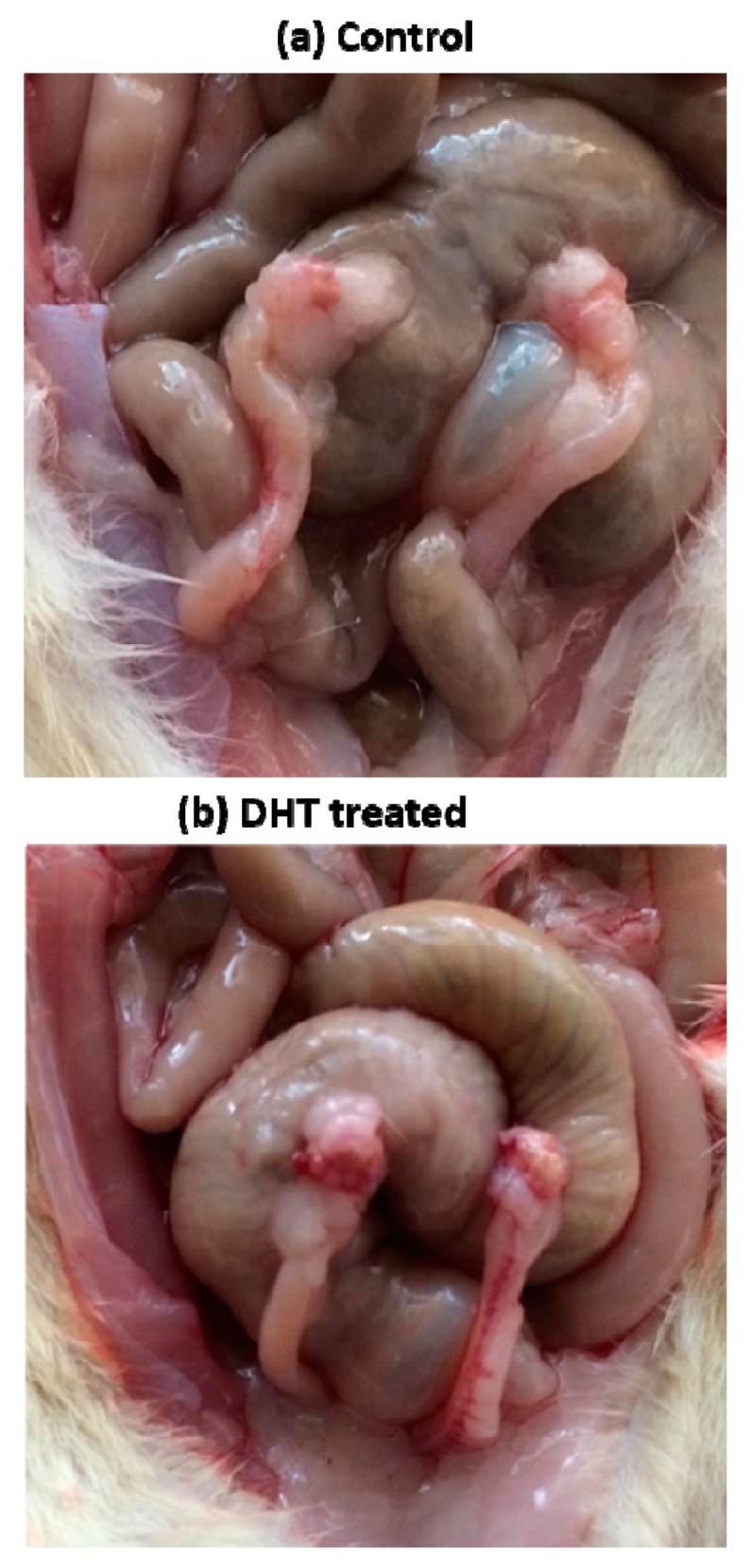
The gross appearance of ovary in (**a**) control and (**b**) dihydrotestosterone (DHT)-treated hyperandrogenised polycystic ovary morphology (PCOM) rat.

As compared with the control group, the hyperandrogenised rats showed non-significant increase in the body weight (Figure 1a). However, the total ovary weight (Figure 1b) and relative ovary weight (Figure 1c) of the DHT-treated group were significantly lower than those of control. The ovarian morphology revealed the presence of multiple cysts (Figure 2b) indicating the presence of PCOS-like phenotype, PCOM. The PCOM phenotype was further confirmed by the statistically significant increase in the follicular cell subtypes and decreased corpus luteum number (Figure 1d).

### 4.2. Biochemical Results

#### 4.2.1. Serum Hormonal Profiles

**Figure 3:**
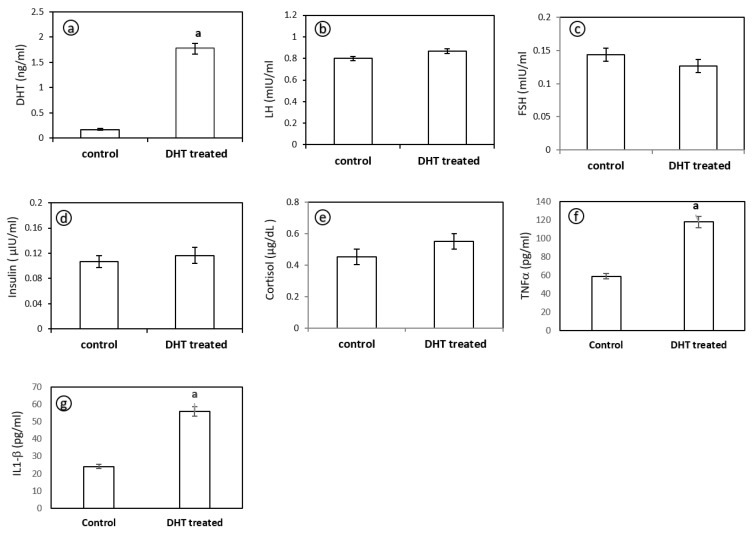
Levels of dihydrotestosterone (DHT) (**a**), luteinizing hormone (LH) (**b**), follicle stimulating hormone (FSH) (**c**), insulin (**d**), cortisol (**e**), tumor necrosis factor-α (TNF-α) (**f**) and interleukin-1β (IL-1β) (**g**) in control and DHT-treated hyperandrogenised PCOM rat. Each bar represents mean ± SD (n = 6). ‘a’ denotes statistical significance at *p* < 0.05 when compared with control.

The DHT levels were higher in hyperandrogenised PCOM rats (1.7-fold) compared to the control rats (Figure 3a). Serum profile showed no significant alterations in hormones namely luteinizing hormone (LH) (Figure 3b), follicle stimulating hormone (FSH) (Figure 3c) and insulin (Figure 3d) between control and PCOM rats. As shown in Figure 3e, there was only a slight elevation in the stress hormone cortisol in hyperandrogenised PCOM rats as compared to control.

#### 4.2.2. Serum Levels of TNF-α and IL-1β

Hyperandrogenised PCOM rats demonstrated significantly elevated levels of TNF-α (Figure 3f) and IL-1β (Figure 3g) compared to control rats indicating a pro-inflammatory condition in hyperandrogenised state.

### 4.3. Histopathological Examination

**Figure 4:**
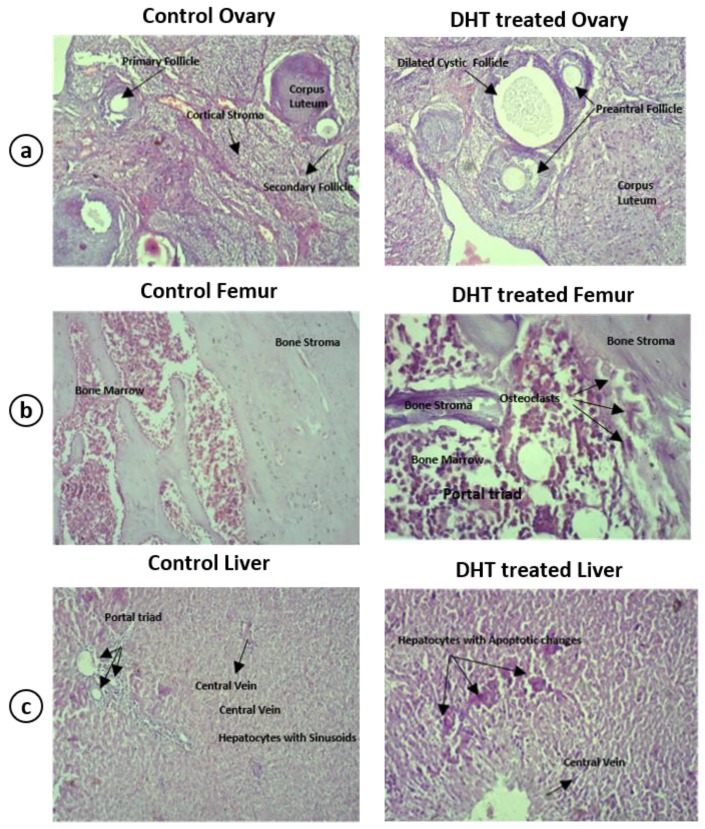
Histopathology of ovary (**a**), femur (**b**) and liver (**c**) in control and DHT-treated hyperandrogenised PCOM rat. Inflammatory cells were evident in the tissue section of DHT-treated PCOM rats indicating the onset of inflammation.

Histopathological observation revealed the onset of inflammation as evidenced by inflammatory cells in the liver, femur, and ovary of hyperandrogenic PCOM rats (Figure 4). The ovary showed multiple dilated follicles, the presence of multinucleated giant cells and lymphocytes indicating the inflammation (Figure 4a) as compared to control. The ovary also had reduced number of corpus luteum, increased number of primary/preantral follicles, along with the presence of large number of cystic follicles as compared to control. The PCOM rat demonstrated increased focal osteoclastic activity in the femur with normal bony cortex and trabeculae (Figure 4b). The liver showed a large number of focal necrotic hepatocytes suggesting inflammation (Figure 4c).

### 4.4. mRNA Expression of Inflammatory Cytokines and Stress-Related Peptides

**Figure 5:**
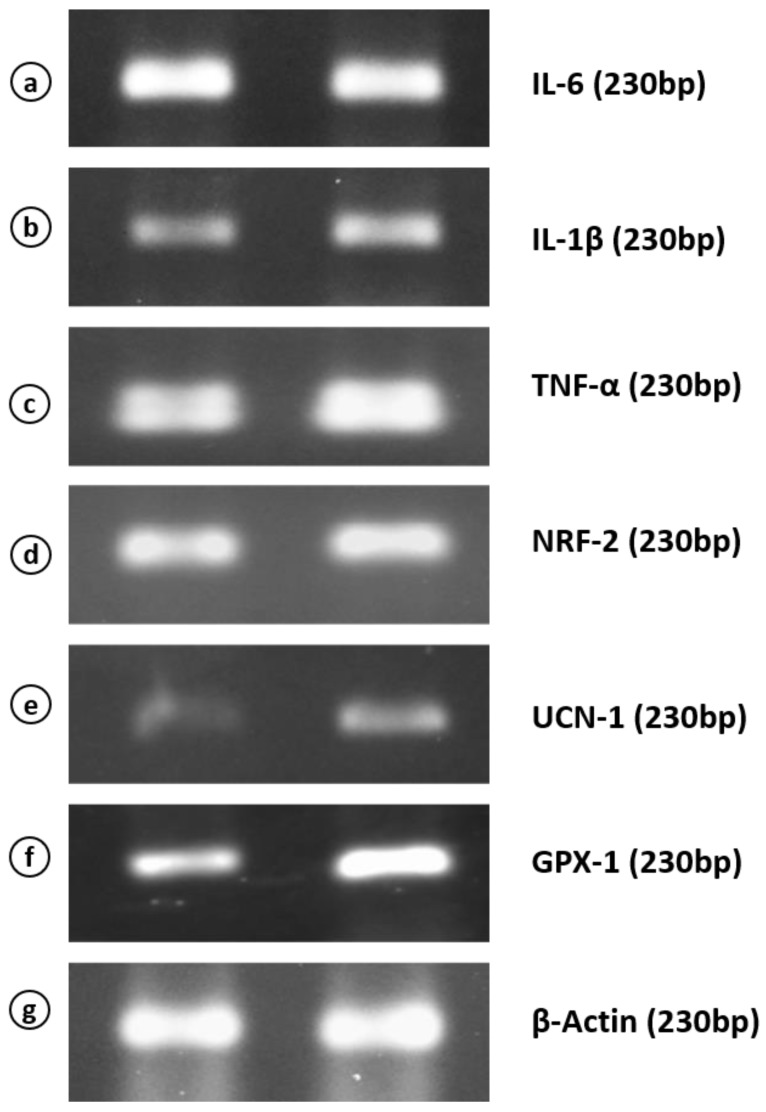
Semi-quantitative PCR analysis of mRNA expression levels of interleukin-6 (IL-6) (**a**), interleukin-1β (IL1–β) (**b**), tumor necrosis factor-α (TNF-α) (**c**), nuclear factor erythroid 2-related factor-2 (Nrf2) (**d**), urocortin-1 (Ucn1) (**e**), glutathione peroxidase-1 (Gpx-1) (**f**) and β-actin (**g**) in the liver of control and hyperandrogenic PCOM rats.

**Figure 6:**
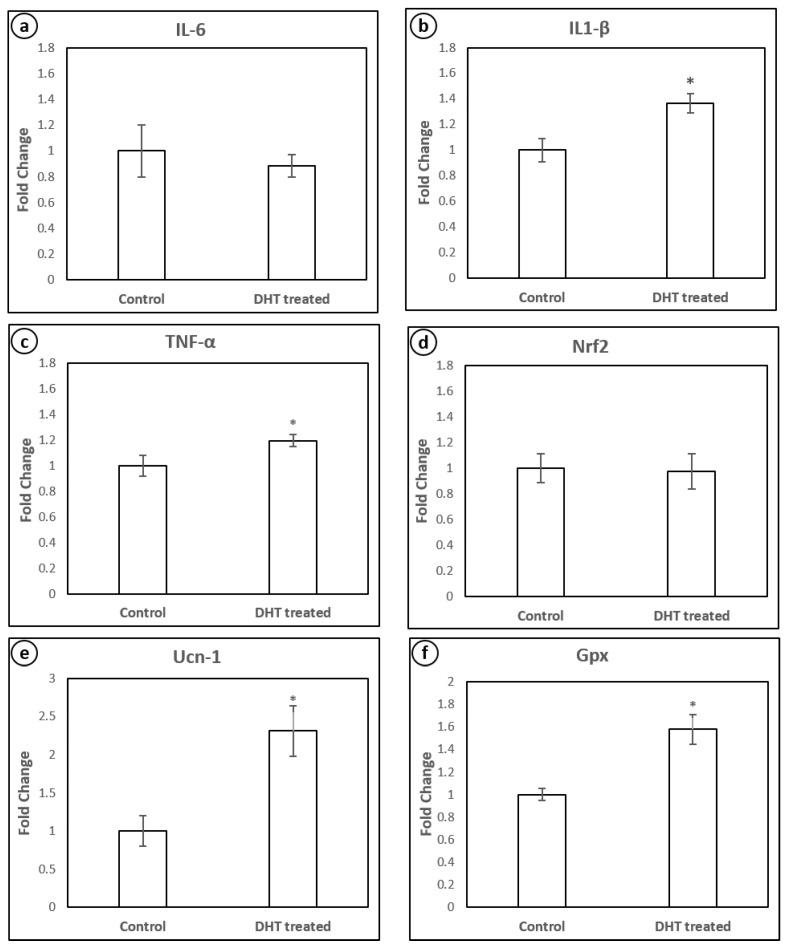
Quantification (fold change) of mRNA expression levels of IL-6 (**a**), IL1-β (**b**), TNF-α (**c**), Nrf2 (**d**), Ucn-1 (**e**) and Gpx (**f**) in the liver of control and hyperandrogenic PCOM rats. Each bar represents mean ± SD (n = 6). ‘*’ denotes statistical significance at *p* < 0.05 when compared with control.

The hyperandrogenised PCOM rat liver did not show any significant difference in interleukin-6 (IL-6) (Figure 5a and Figure 6a) and nuclear factor erythroid 2-related factor-2 (NRF-2) mRNA levels (Figure 5d and Figure 6d), but showed a significant upregulation in the expression level of inflammatory cytokines TNF-α (Figure 5c and Figure 6c) and IL-1β (Figure 5b and Figure 6b) levels as compared to control. The expression of stress-related protein urocortin 1 (Ucn-1) (Figure 5e and Figure 6e) and the antioxidant gene glutathione peroxidase-1 (Gpx1) (Figure 5f and Figure 6f) expression was highly upregulated in the liver during hyperandrogenised PCOM state.

## 5. Discussion

PCOS induction in rats is performed through several methods including physical manipulations, genetic modifications and by using various androgenic hormones. Some of the common methods for inducing PCOS in rats include constant light exposure, hypothalamic lesions, administration of testosterone propionate (TP), testosterone (T), dehydroepiandrosterone (DHEA), androstenedione, dihydrotestosterone (DHT), administration of estrogen in early postnatal life and anti-progesterone and aromatase inhibitor letrozole [22,23]. In this study, we used DHT, a potent non-aromatizable androgen for induction of PCOS-like phenotype in 21-days-old prepubertal female albino rats. Rats were implanted with an osmotic pump designed to release 3.46 μg DHT/h at a daily dose of 83 μg for 90 days. After 90 days, the rats showed irregular estrous cycles as evidenced by the presence of cornified cells (data not shown) indicating anovulation and ovarian features mimicking PCOS [24]. Administration of androgen to female rats during early postnatal life resulted in polyfollicular anovulatory ovaries during puberty and adulthood [25,26]. The present study evidenced the onset of cysts in the ovaries (Figure 2b), which is corroborated with histopathological observations (Figure 4a) that attest to the utility of the model system employed. The histopathology of ovary of DHT-treated rats displayed multiple dilated follicles and numerous corpus luteum. The observations are associated with the presence of necrotic hepatocytes as evident by histological findings of liver microarchitecture (Figure 4c). Therefore, we presume the onset of inflammation by a continuous release of DHT could have exerted the histoarchitectural changes in the liver of DHT-treated rats. To address the notion, we estimated the levels of serum levels of DHT, LH, FSH, insulin, cortisol (Figure 3a–e), TNF-α and IL-1β (Figure 3f,g) in control and DHT-treated PCOM rats. The present study reported no change in the levels of insulin, which indicated that it is not regulated by exogenous administration of DHT. The existence of non-insulin resistance PCOS is reported earlier as a clinical and endocrinological subgroup [27,28,29]. Shah [30] reported that nearly 60% of the patients (101 out of 167) with non-insulin resistance PCOS, which further supported the present findings. Therefore, the results of the present study could be regarded as non-insulin resistance PCOS. Earlier reports showed the unaltered levels of LH [18,31], FSH [18,31,32,33] and elevated levels of T/DHT [18,34,35,36]. The present investigation showed unaltered levels of LH, FSH, and elevated level of DHT further supported the fact that the elevated LH/FSH may not be a key factor causing the PCOM phenotype. A significant increase in the level of TNF-α and IL-1β (Figure 3f,g) provided solid support to the pro-inflammatory effect of enhanced DHT on PCOM rats.

For effective local and systemic homeostasis of inflammation, the immune cells residing in the liver along with other non-hematopoietic cells play a crucial role. Maintenance of an optimal cytokine milieu is a prerequisite for the liver to execute its physiological function [37]. Any disturbances in this homeostasis can thus lead to elevated cytokines resulting in various inflammatory disorders. To address the regulatory role of DHT on the expression pattern of inflammatory cytokines, we evaluated the mRNA expression pattern of TNF-α, IL-1β, and IL-6.

Though there is considerable knowledge regarding the cellular and molecular pathways of the inflammatory process, much remains to be understood, including the role of sex steroid hormones in promoting or attenuating inflammatory responses. Several epidemiological, as well as clinical studies, show that androgens attenuate the expression of inflammatory biomarkers including TNF-α, IL-1β, and IL-6 in various chronic inflammatory diseases like Crohn’s disease, psoriasis, rheumatoid arthritis and allergic asthma [38]. Androgens regulate a variety of molecular pathways involving a host of immune cells and biochemical factors that contribute to the regulation of the inflammatory process [39]. Various mechanisms are postulated to suggest the role of androgen in regulating the production of inflammatory molecules including its effect on the expression of toll-like receptor-4 (TLR-4), neutrophil-binding vascular adhesion molecule-1 (VCAM-1), suppression of monocyte chemoattractant protein-1 (MCP-1), IL-6 expression in 3T3-L1 adipocytes, etc. [38,40,41]. During wound healing, androgens have been shown to increase the production of pro-inflammatory cytokines by macrophages. After traumatic hemorrhagic shock and burns, androgens also inhibited the production of cytokines [38]. All these findings suggest that androgens are necessary for maintaining overall inflammatory homeostasis.

Androgen receptors (ARs) are widely expressed in many endocrine organs and key metabolic tissues including the liver [42,43]. Androgen signaling in macrophages has demonstrated roles in key cellular functions including chemotaxis and cytokine secretion [44]. The liver consists of several different cell types such as Kupffer cells, Ito cells, lipocytes, hepatocytes, and fibroblasts. Kupffer cells sense the immunological response through TLR and complement receptors and Fc-receptors. Kan et al. [45] reported that a reduction in the systemic inflammatory response by flutamide prevented liver injury. Thus, liver Kupffer cells and androgen/ARs play an important role in maintaining immune response homeostasis [46].

A significant increase in the serum of TNF-α and IL-1β reported in the present study indicates a direct role of DHT in inducing inflammatory genes. A recent study has reported that rats exposed to DHT and insulin suffer mitochondrial damage, which establishes oxidative stress in the uterus [47]. More importantly, androgen is reported to recruit monocytes and macrophages in the ovaries during the onset of PCOS [48]. Androgen-induced apoptosis in granulosa cells was shown to be mediated by macrophages. A recent observation has indicated the changes in the immunological state of various organs such as blood, spleen, and kidney during hyperandrogenism [49].

The role of TNF in mediating innate and adaptive immune responses is known [50]. A variety of immune cells such as monocytes, lymphocytes, neutrophils, macrophages, and dendritic cells are regulated by TNF. Even inflammation, infection and stress are known to induce the expression of TNF-α and regulate diverse immune functions. In addition, antigen presentation by T-cell pattern recognition receptors is known to influence the expression of TNF-α. Many of the positive regulators of TNF-α are shown to activate the transcription factor nuclear factor of activated T-cells (NFAT) and promote the transcription of TNF-α. These transcription factors are dephosphorylated through calcineurin, a calcium-dependent phosphate that facilitates the nuclear translocation of NFAT [51]. In this regard, it is pertinent to mention a study that showed the increase in NFAT activity by testosterone and modulation of its transcriptional activity [52].

The production of IL-1β by immune cells is considered important to execute inflammatory responses [53]. During inflammation stimulation, IL-1β is secreted and released into the bloodstream, where it exerts influence on other cells [54]. Activation of NFAT5 is known to induce the expression of TNF-α and IL-1β, as the levels of these cytokines were greatly reduced upon NFAT5 inhibition [55]. We have already discussed the role of AR-signaling in the activation of NFAT. Thus, though anti-inflammatory in nature, androgen may exert a pro-inflammatory effect in PCOS condition by aiding the secretion of inflammatory cytokines, mainly TNF-α and IL-1 β via the transcriptional activation of NFAT.

In the present investigation, it is observed that Ucn-1 mRNA transcript level is stupendously elevated in the liver of PCOM rats. Urocortin, a stress-related protein, has major regulatory effects on both reproductive and immune function [56], and is found to be involved in both pro-inflammatory and anti-inflammatory effects in various tissues.

Urocortin is reported to be involved in the activation of MAPKs and PI3K/AKT pathways and also cAMP signaling pathways, which play a critical role in cytokine production including TNF-α after LPS stimulation [7]. Studies show that in certain inflammatory disorders like colitis, there is an upregulation in the expression of urocortin mRNA [7,56]. TNF-α, angiotensin II, lipopolysaccharides, pyrrolidine dithiocarbamate and H_2_O_2_ are some of the positive regulators of urocortin [57]. Androgen may influence these regulators resulting in altered urocortin expression [58]. A link between NAFLD and inflammation is reported as a common liver disorder wherein a crucial role for liver macrophage (Kuffer cell) is reported [59]. It is also reported that high plasma Ucn1 levels were reported in endometriosis patients [60]. In this context, the identification of urocortin during an inflammatory state in the liver and hyperandrogenism opens a new avenue on the role of androgens in the regulation of urocortin. It is suggested that a balanced level of urocortin expression is required for maintaining inflammatory homeostasis [61]. The present investigation indicates the relevance of urocortin as a therapeutic target for inflammatory disorders like PCOS.

The findings of the present investigation assume a great significance, as there is an increasing link between NAFLD and PCOS. Schwimmer et al. [62] reported elevated levels of alanine aminotransferase (ALT) activity in 30% of 70 infertile women with PCOS, who do not have family history or other factors that would predispose them to liver diseases. High levels of fasting insulin along with alteration in aminotransferase activity are reported in subjects with PCOS with NAFLD [63]. Gambarin-Gelwan et al. [64] identified fatty liver in 55% of women with PCOS. They proposed high BMI and insulin resistance as the two main risk factors associated with fatty liver in the PCOS subjects. In another study, it was shown that the elevated biomarkers, serum ALT and gamma-glutamyltransaminase (GGT) associated with NAFLD in obese PCOS women could be attenuated or reversed using metformin therapy, suggesting the role of insulin resistance [65]. Elevated ALT levels of NAFLD in PCOS were positively correlated with free androgen index (FAI) and total testosterone levels [66]. PCOS, NASH and metabolic syndrome are inter-linked via the presence of insulin resistance. During insulin resistance, the production of IL-6 and TNF-α is increased in fat cells and they contribute to intra-abdominal excess fat.

The elevated levels of inflammatory cytokines in patients with nonalcoholic steato hepatitis (NASH) and PCOS are reported [67]. Vassilatou et al. [13] reported that bioavailable androgens might be implicated in the pathogenesis of NAFLD in women with increased total androgen concentrations. Abdominal adiposity and more severe dyslipidemia are also implicated in the NAFLD in women with PCOS. They point out the importance of evaluating patients with PCOS for NASH in an attempt to reverse it to minimize its effects on liver dysfunction and the development of cirrhosis. Tan et al. [54] found increased hepatic apoptosis in PCOS patients reflected by elevated M30 levels. They suggest that hyperandrogenism and metabolic disturbances such as obesity, hyperglycemia, and dyslipidemia seem to be only secondary, possibly via insulin resistance (IR), connected with hepatic apoptosis. Their data show that PCOS represents a risk factor for NASH progression with long-term consequences, including the risk for fibrosis, cirrhosis, and hepatocellular carcinoma. They suggest that NASH should be considered in PCOS patients independent of liver enzyme levels, especially when IR is present. The high fatty liver index is a common feature among PCOS women with obesity and correlated with metabolic syndrome [68]. Thus, PCOS women are at high risk of liver disease. The possible risk factors associated include insulin resistance, obesity, and hyperandrogenism. The exact mechanism behind the development of liver disease in PCOS is of key importance to avoid the complications associated with it.

## 6. Conclusions

The results obtained in the present study provide evidence for the pro-inflammatory role of non-aromatizable androgens in female rats. The study also reports the enhanced gene expression pattern of urocortin in the liver of the hyperandrogenism animal model of PCOM. This study also delineates the link between the endocrine and immune system in PCOM rats, which could be responsible for the chronic inflammatory state among them. The results obtained in the present study could also find potential application in conditions such as hyperprolactinemia and non-classic congenital hyperplasia. Future studies should be aimed at simulating hyperandrogenism in the cell culture model system to unravel the regulatory role of DHT on inflammation, which is currently being pursued in our laboratory. The present study also highlights the necessity to consider drugs that target inflammatory cells and exert an ameliorative impact on the immune cells of PCOS subjects.

## Figures and Tables

**Table 1:** Primer Sequences Used in the Present Study.

S.No	Gene	Forward Primer	Reverse Primer	Accession Number	Product Size
1	IL-6	TGATGGATGCTTCCAAACTG	GAGCATTGGAAGTTGGGGTA	NM_012589.2	230
2	IL-1 β	CACCTTCTTTTCCTTCATCTTTG	GTCGTTGCTTGTCTCTCCTTGTA	NM_031512.2	241
3	TNF-α	AAATGGGCTCCCTCTCATCAGTTC	TCTGCTTGGTGGTTTGCTACGAC	XM_008772775.2	111
4	Nrf2	CACATCCAGACAGACACCAGT	CTACAAATGGGAATGTCTCTGC	NM_031789	121
5	Ucn-1	CTCCTGGTAGCGTTGCTGCTTCTG	GCCCACCGAATCGAATATGATGC	NM_019150.1	339
6	Gpx	GTCCACCGTGTATGCCTTCTCC	TCTCCTGATGTCCGAACTGATTGC	NM_030826.4	218
7	β-actin	AGCCATGTACGTAGCCAT	CTCTCAGCTGTGGTGGTGAA	NM_031144.3	228
